# Raman Spectroscopic Measurements of Dermal Carotenoids in Breast Cancer Operated Patients Provide Evidence for the Positive Impact of a Dietary Regimen Rich in Fruit and Vegetables on Body Oxidative Stress and BC Prognostic Anthropometric Parameters: A Five-Year Study

**DOI:** 10.1155/2016/2727403

**Published:** 2016-04-26

**Authors:** A. Perrone, A. M. Pintaudi, A. Traina, G. Carruba, A. Attanzio, C. Gentile, L. Tesoriere, M. A. Livrea

**Affiliations:** ^1^Dipartimento STEBICEF, Università di Palermo, 90123 Palermo, Italy; ^2^Department of Oncology, ARNAS Ospedali Civico e Benfratelli G. Di Cristina e M. Ascoli, 90127 Palermo, Italy

## Abstract

Dermal carotenoids are a feasible marker of the body antioxidative network and may reveal a moderate to severe imbalance of the redox status, thereby providing indication of individual oxidative stress. In this work noninvasive Resonance Raman Spectroscopy (RRS) measurements of skin carotenoids (skin carotenoid score (SCS)) were used to provide indications of individual oxidative stress, each year for five years, in 71 breast cancer (BC) patients at high risk of recurrence. Patients' SCS has been correlated with parameters relevant to BC risk, waist circumference (WC), and body mass index (BMI), in the aim of monitoring the effect of a dietary regimen intended to positively affect BC risk factors. The RRS methodological approach in BC patients appeared from positive correlation between patients' SCS and blood level of lycopene. The level of skin carotenoids was inversely correlated with the patients' WC and BMI. At the end of the 5 y observation BC patients exhibited a significant reduction of WC and BMI and increase of SCS, when strictly adhering to the dietary regimen. In conclusion, noninvasive measurements of skin carotenoids can (i) reveal an oxidative stress condition correlated with parameters of BC risk and (ii) monitor dietary-related variations in BC patients.

## 1. Introduction

A proper redox balance is essential to control signalling pathways governing cell functions, including those known to influence cell proliferation [1–4]. Indeed critical variations of the redox homeostasis, not timely compensated by endogenous systems, result in oxidative stress and concur to dysfunction of several regulatory mechanisms leading to cancer onset and development. Dietary plant constituents are now acknowledged to play a significant role in this context. The intense interaction of redox-active phytochemicals with cells at different stages of cancer development is considered essential to maintain the redox balance governing pathways that control proliferation and evasion of cell-death, thus contrasting both cancer onset and evolution [5, 6]. All this has appeared strongly supported by epidemiological observations providing clear evidence that a dietary pattern such as the traditional Mediterranean way of eating, including large amounts of plant foods and derivatives, that is, fruits, vegetables, olive oil, red wine, and legumes, may play a primary role in promoting health and preventing onset and progression of chronic and degenerative disorders including various types of cancer [7–13].

Measuring the concentration of antioxidants in blood samples may provide indication on the individual capacity to maintain an optimal redox balance or conversely reveal an oxidative stress status. Molecular antioxidants in the body work in concert and preserve each other; therefore the serum level of each one, including carotenoids, is predictive of the level of all others [14, 15] and is an expression of the individual antioxidant status. An optical method, based on the Resonance Raman Spectroscopy (RRS), has recently been developed for a noninvasive measurement of carotenoids in human tissues. RRS is a laser spectroscopy based on the Raman effect. When a low intensity laser monochromatic light interacts with some molecules, these diffuse the light emitting a new, higher wavelength, monochromatic light [16] that can be revealed by a scanner converting Raman intensity in counts. Because of their conjugated carbon backbone, carotenoids possess characteristic vibrational/rotational energy levels that make them particularly well suited for RRS, strongly absorbing in the blue wavelength region and emitting in the green one. RRS methodology has successfully been exploited for the quantitative measurement of carotenoids in human macula lutea, oral mucosa, and skin [17–20]. RRS measurements of carotenoids in skin have been validated by comparing with extraction and conventional high performance liquid chromatography measurements of carotenoids in skin samples [21]. Importantly, the level of dermal carotenoids has appeared significantly correlated with the blood carotenoid level [22–24]. In this context, the amount of dermal carotenoids revealed by RRS can be considered a marker of the individual antioxidative network, and its measurement was applied to assess the body redox state and eventually provide evidence of critical conditions in diseases. Indeed, RRS measurements in humans have been inversely correlated with urinary isoprostanes, known biomarkers for oxidative stress [25].

RRS has been applied in our recent investigation aimed at monitoring oxidative stress of beta-thalassemia patients and correlating the amount of skin carotenoids with iron overload [26].

In this work that comes as a complementary study of a clinical trial starting in 2009 and ending in 2014, RRS measurements of skin carotenoids have been carried out in breast cancer (BC) operated patients, to research the oxidative stress associated with this condition and monitor eventual variations from a five-year-long treatment aimed at reducing BC risk and recurrence by combining conventional therapies with dietary intervention [27, 28]. The latter was based on a traditional Mediterranean-style regimen, where a high daily consumption of fruit and vegetables was fundamental and was associated with a moderate physical activity. To substantiate the RRS approach the level of dermal carotenoids has been correlated with markers of BC risk (waist circumference, WC, and body mass index (BMI)) [29], which finally provided evidence of the effectiveness of the RRS measurements to monitor patient compliance and influence of the diet.

## 2. Patients and Methods

### 2.1. Subjects and Protocol

DIANA5 (Diet and Androgens-5) is an Italian multicenter project of alimentary education, aimed at preventing BC recurrence in patients surgically treated for BC in the previous 5 years, who have not developed distant metastasis or second primary BC and are at high risk based on their hormonal and/or metabolic milieu; that is, they exhibit one or more of high risk traits (metabolic syndrome, oestrogen receptor negative tumor, and high serum testosterone or insulin level) and associated abnormal anthropometric parameters, WC and BMI [28]. Clinicopathological measurements, including blood level of sex hormones, insulin, IGF-1, oestrogen receptors, glucose, and lipid parameters, are determined as reported [30] to serve as inclusion criteria in the project and are monitored at baseline and yearly to detect eventual changes during the time of intervention.

BC patients have been enrolled at the Department of Oncology, ARNAS Ospedali Civico e Benfratelli G. Di Cristina e M. Ascoli, Palermo, Italy, in January 2009, and followed up to the end of 2014. Apart from conventional therapies, patients were treated with dietary intervention. This was based on a traditional Mediterranean diet, prescribed consumption of seasonal fruits and vegetables (F/V, 5 servings a day), unrefined grains, legumes, and olive oil, whereas sugared drinks, alcoholic beverages, processed meat, and animal fats were forbidden (detailed description of diet has been reported in [30]). The intervention was intended to decrease level of sex hormones, insulin and insulin growth factor 1 (IGF-1), and reduce risk factors associated with BC prognosis, BMI and WC, [31, 32], additional breast cancer events, and the risk of metastasis [11]. Moderate physical activity was also recommended. All patients received the WCRF-based guidelines for cancer prevention (see the list below) and were invited to participate in dedicated kitchen courses and physical exercise sessions; in addition, they filled in a questionnaire reporting on their own life- and dietary-style. The patients were followed up for vital status and BC related events, including BC-specific mortality, distant metastasis, local recurrences, and contro-lateral BC, which were obtained by self-reporting every six months throughout the study.

The following list is based on the World Cancer Research Fund (WCRF) and American Institute of Cancer Research (AICR). 

*WCRF/AICR 2007 Recommendation*

- Be as lean as possible within the normal range of body weight.
- Be physically active as part of everyday life.
- Limit consumption of energy-dense food and avoid sugary drinks.
- Eat mostly food of plant origin, with a variety of nonstarchy vegetables and fruit every day and unprocessed cereals and/or pulses within every meal.
- Limit intake of red meat and avoid processed meat.
- Limit alcoholic drinks.
- Limit consumption of salt and avoid mouldy cereals or pulses.
- Aim to meet nutritional needs through diet alone.
- Children to be breastfed by their mothers for at least six months.
- Cancer survivors follow the recommendation for cancer prevention.

### 2.2. The Carotenoid Study

In 2009, a number of BC patients (*n* = 71) were randomly recruited among the patients enrolled at the Centre of Palermo, aged 36 to 74, nonsmokers, accepted with informed consent, and who were to be examined for body redox status, and were invited for RRS measurement of skin carotenoids (skin carotenoid score (SCS)), as an index of oxidative stress. Each patient contributed a duplicate measurement at the baseline and then each year, with a 12-month interval, until 2014. Anthropometric measurements, weight (kg), and WC (cm) were collected each time, and BMI (body mass in kilograms/square of height in meters, kg/m^2^) was measured as reported [29]. Our observation ended in June 2014 and all patients terminated the study.

Healthy women (HW) were interviewed and examined for skin carotenoids in 2009. The mean SCS from a number of nonsmoking HW (*n* = 120), reporting being used to eating high amounts of F/V (5 servings a day), aged 38 to 70, BMI between 20.5 and 31 (mean 24.5 ± 3), and WC between 62 and 100 (mean 80.6 ± 6.3), was taken as the reference SCS throughout our study (HW-SCS, 39,210 ± 9,400; min HW-SCS 20,000; max HW-SCS 75,000).

### 2.3. Measurement of Carotenoids in Skin

A portable Raman spectroscope, Pharmanex® BioPhotonic Scanner S2 (NuSkin, Provo, Utah, USA), designed to monitor carotenoids in the 0.1 mm stratum corneum of the skin of the hand, has been used for the measurements. A low intensity 471.3–473 nm radiation from light emitting diodes interacts with the skin carotenoids. The scattered light is detected at 507.8–509.8 nm by the scanner that converts the Raman intensity in counts (skin carotenoid score (SCS)). A computer then transforms the scanner signals in a colored scale going from red (poor carotenoid score, <19,000) to dark blue (high carotenoid score, >50,000). SCS can be converted to laboratory measurements using the equation [*Y* = 12703 × *X* + 5891.7], where “*Y*” is the SCS value and “*X*” is the carotenoid concentration expressed as micrograms/mL of serum.

### 2.4. Measurement of Lycopene

Blood lycopene of patients who submitted themselves to skin carotenoid evaluation was measured. Duplicate measurements from the same sample were carried out. Lycopene was extracted from 500 *μ*L serum samples, diluted 1 : 2 with 0.15 mM NaCl, with 1 volume of methanol and 3 volumes of hexane: diethyl ether (l : l, vol : vol). The extracts were then dried under nitrogen, resuspended with a mixture of acetonitri1e : methanol : tetrahydrofurane (58.5 : 35 : 6.5, vol : vol : vol), and analyzed with the same solvent [33] by a HPLC Supelco Supelcosil LC-18 column (0.46 × 25 cm) (Bellefonte, PA), at a flow rate of 2.5 mL min^−1^. Under these conditions lycopene eluted at 8.2 minutes. Revelation was at 450 nm.

### 2.5. Statistical Analysis

BMI, WC, and SCS are expressed as means and standard deviation (SD) of the patients' values. Differences in SCS among groups of fruit and vegetable intakes and BMI were tested by analysis of variance (ANOVA) and Bonferroni post hoc tests, with *p* < 0.05 being taken as significant. Pearson's correlations were used to determine the relationship between SCS and BMI or WC. All statistical analyses were done using GRAPHPAD PRISM v5 (GraphPad Software, San Diego, California, USA), SYSTAT version 10.0 (SPSS, Chicago, IL, USA), and Microsoft Excel.

## 3. Results and Discussion

Cancer control can be achieved by decreasing the rate of oxidative stress and enhancing antioxidant defense mechanisms. In this context the role of F/V and dietary redox-active phytochemicals in reducing the risk of cancer including BC [11, 27, 30, 34], by regulating antioxidant defense mechanisms and redox signaling, has long been described [35–37]. Dermal carotenoids can be regarded as biomarkers of the body antioxidant status [16, 38], as well as of fruit and vegetable intake in nutritional studies [21]. On this basis, dermal carotenoids have been monitored for five years in 71 BC operated patients at high risk of recurrence, to assess oxidative stress status and influence of dietary treatment based on high daily consumption of fruit and vegetables.

### 3.1. Dermal Carotenoids Are Correlated with Plasma Lycopene in BC Patients

**Figure 1:**
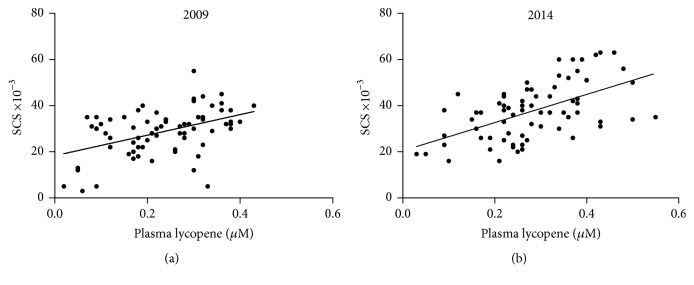
Correlation between plasma lycopene and skin carotenoid score (SCS) in BC patients (*n* = 71): (a) before (*r* = 0.450; *p* < 0.0001) and (b) after (*r* = 0.559; *p* < 0.0001) five years of dietary intervention. SCS and lycopene values are the mean of duplicate measurements (*n* = 71; *p* < 0.0001).

Relatively high concentrations of carotenoids accumulate in human skin. The RRS-based measurement of dermal carotenoids has been validated in healthy adults by pairing blood and skin levels of carotenoids [21]. Feasibility of the RRS approach to measure skin carotenoids as a reflection of their plasma concentration was at first explored in BC patients. Since lycopene is the major carotenoid in the skin [17, 18], the individual correlation between SCS and the plasma level of lycopene was examined.  Figure 1(a), reporting data at the baseline in 2009, shows a net positive correlation, thus validating the use of this spectroscopic method to evaluate the body carotenoid level even in BC patients. The positive correlation was also confirmed at the end of the observation in 2014 (Figure 1(b)).

### 3.2. Variations of Dermal Carotenoids in BC Patients during the 5 y Dietary Intervention

**Figure 2:**
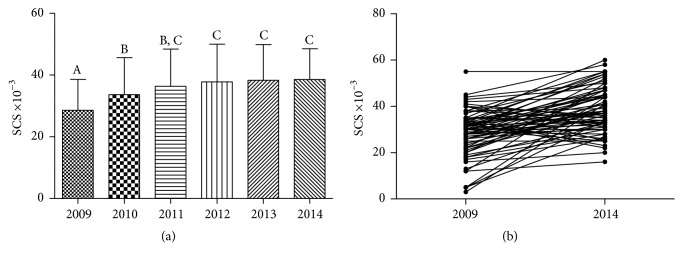
Skin carotenoid score (SCS) of BC patients (*n* = 71) during the five years of dietary intervention. (a) Data are the mean ± SD of measurements carried out in duplicate on the patients once a year. Values of columns labelled with different letters are statistically different (*p* < 0.05, Anova one way followed by Bonferroni's multiple comparison test). (b) Values of single patients at starting (2009) observation and at the end (2014) of observation.

To the best of our knowledge, only a few trials investigated skin carotenoid response by Raman Spectroscopy to controlled diets [39] including increase of fruit and vegetables [24, 40], and none was carried out on cancer patients. BC patients underwent dermal carotenoids measurements with a 12-month interval to assess the evolution of the oxidative stress status during the 5 years of observation. SCS (means ± SD of the values recorded at the end of each year) are reported in Figure 2(a). The SCS increased significantly during the first and second year of observation; thereafter only a nonsignificant positive trend was observed (Figure 2). However, while the SCS of patients at starting (28,580 ± 10,060, *n* = 71) was approximately 28% lower than the reference value of healthy women (HW-SCS = 39,210 ± 9,400, *n* = 120) (*p* < 0.001, Student's *t*-test), no significant difference was found at the end of observation (mean SCS 38,590 ± 9,920, *n* = 71) (*p* = 0.67), indicating improvement of the antioxidant status. On the other hand, a very high interindividual variability in the SCS progress and evolution was evident when the values of each patient in 2009 (*n* = 71) were judged against the relevant measurements 5 years later (Figure 2(b)): the increase varied from 101% to 1300% (*n* = 56); 3 of the patients did not change their level of carotenoids, and 12 of them exhibited a decrease varying between 3% and 30%. It may be interesting to mention that the major percent increments were observed in patients showing the highest redox imbalance at starting (*n* = 9, SCS 3,000 to 15,000), whereas the patients exhibiting a decrease of the carotenoid score were in a medium-high SCS range (30,000 to 40,000). A number of reasons including adiposity [16] and/or concomitant therapies may account for the differences between the individual response to the intervention, including a higher or lower adherence of the patients to the dietary rules.

### 3.3. Variations of BMI and WC in BC Patients before and after the 5 y Dietary Intervention

**Figure 3:**
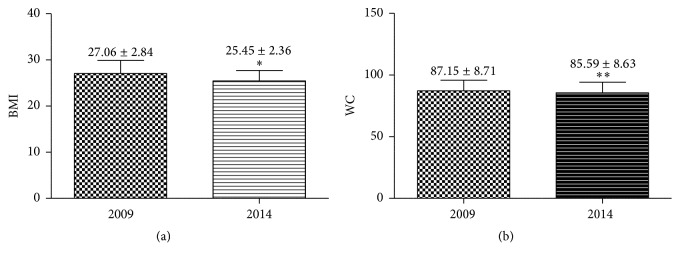
Body mass index (BMI) (a) and waist circumference (WC) (b) of BC patients at starting (2009) and at the end (2014) of five years of dietary intervention. Values are the mean ± SD of measurements carried out on all patients (*n* = 71). With respect to the relevant values in 2009, values are significant with ^*∗*^ *p* < 0.0001 or ^*∗∗*^ *p* = 0.0072 (paired *t*-test).

The importance of BMI and WC as prognostic factors in BC is well documented [31, 32]; then measurements of these parameters can help to follow the evolution in the status of BC patients. The Mediterranean dietary pattern is intended to reduce risk factors for breast cancer and then affect positively BMI and WC [41]. This was also observed in the patients under our observation.  Figure 3 compares the mean values of BMI and WC in patients in 2009 and at the end of the intervention in 2014. Both BMI and WC were significantly reduced.

### 3.4. Correlation between Oxidative Stress and BC Risk Factors

**Figure 4:**
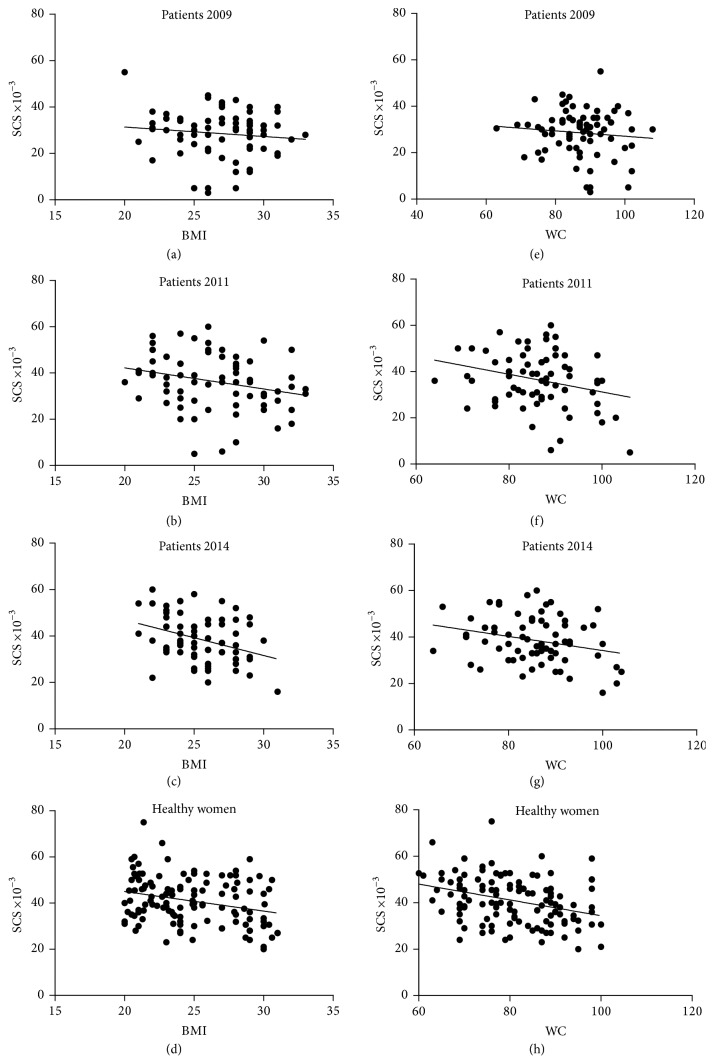
Correlation between SCS and either BMI (a–c) or WC (e–g) in BC patients (*n* = 71) at starting and during the course of five years of dietary intervention and in healthy women (*n* = 120, (d) and (h)). Each SCS value is the mean of duplicate measurements. Patients: (a) (*r* = −0.118; *p* = 0.326); (b) (*r* = −0.259; *p* = 0.028); (c) (*r* = −0.351; *p* = 0.0027); (e) (*r* = −0.099; *p* = 0.408); (f) (*r* = −0.267; *p* = 0.023); (g) (*r* = −0.265; *p* = 0.025); healthy women: (d) (*r* = −0.276; *p* < 0.0022); (h) (*r* = −0.33; *p* < 0.0002).

Since both oxidative stress and anthropometric parameters, either WC or BMI, are associated with the BC pathology and changed positively at the end of the trial, we hypothesized that a correlation existed between these factors. The correlation between SCS and either BMI or BC is shown in Figure 4. Only a negative trend existed at starting (Figures 4(a) and 4(d)), whereas a significant inverse correlation was observed two years later (Figures 4(b) and 4(e)) and at the end of the intervention in 2014, with an even greater significance (Figures 4(c) and 4(f)), indicating that a higher number of patients had increased SCS, that is, achieved a better control of redox status and reduced oxidative stress, with concomitant reduction of BMI and WC. The poor correlation at starting may be an expression of particular additional factors besides the BC status, concurring to an oxidative stress high or very high (e.g., some of the patients had undergone recent surgery and chemotherapy (*n* = 4) and some had been smoker before starting the trial (*n* = 8), which prevents a clear correlation of SCS with BMI and/or BC). The inverse correlation observed after an equilibrium condition was getting on is further indicative of the great importance of a good redox balance and low WC and BMI in the BC pathology. Indeed, a negative correlation between SCS and BMI and WC was also observed in HW (Figures 4(d) and 4(h)), in accordance with data reported for healthy adults [21, 42].

### 3.5. Oxidative Stress and Compliance with Dietary Rules

**Figure 5:**
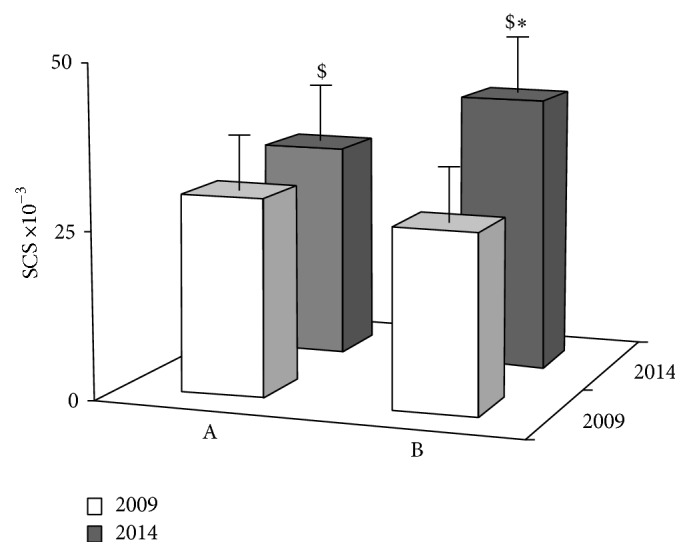
Variations of SCS after five years of dietary intervention in BC patients that had consumed less than five (A, *n* = 39) or five (B, *n* = 32) F/V servings per day. The values are the mean ± SD of duplicate measurements at starting (white bar) and at the end (grey bar) of observation. ($) with respect to the relevant value in 2009, significant with *p* < 0.0001 (paired *t*-test). (*∗*) with respect to A in 2014, significant with *p* < 0.0001 (unpaired *t*-test).

Whether and/or to what extent compliance with the dietary intervention was involved in improving the antioxidant balance was evaluated. At the end of the intervention in 2014, all questionnaires filled in by patients, yearly reporting on their own life- and dietary-style, were examined. Patients were then divided into two groups according to whether they reported to have strictly adhered to the dietary rules and constantly consumed five F/V servings per day (*n* = 39) or whether they had not been steady in observing constantly the dietetic pattern (*n* = 32). The SCS values measured for both groups at the end of observation were then compared with values at starting. The data as means ± SD are reported in Figure 5. At the end of the study, SCS was remarkably lower for the patients that had less than five servings per day, with respect to patients having five, although both groups of patients did not differ significantly at starting (Figure 5). While showing that the better was the compliance of patients with the dietetic pattern, the higher was the body level of antioxidants; these data suggested that diet had a major role in ameliorating their body redox balance and then positively affected oxidative stress.

### 3.6. Follow-Up

Our observation stopped in June 2014. The patients were followed up for vital status and occurrence of new BC events by clinicians. We were informed that, within December 2014, one patient out of 71 who underwent the SCS measurements passed away and five patients had recurrence. We may also report that 43 of all patients recruited at the centre in Palermo (*n* = 391) had recurrence, whereas 13 passed away. A thorough knowledge and analysis of data gathered from all Italian centres participating in the DIANA5 project are necessary to determine to what extent the dietetic pattern and lifestyle adopted may affect the outcome of BC patients.

## 4. Conclusions

The development of biomarkers for oxidative stress as a diagnostic, prognostic, and therapeutic approach has attracted a lot of interest recently. This was the only study designed to evaluate the antioxidant balance as an index of oxidative stress in BC operated patients at high risk of recurrence, by RRS measurement of dermal carotenoids, and monitor the skin carotenoid response to a 5 y long dietary intervention intended to decrease BC risk factors. The treatment indeed caused a decrease of BMI and WC, while the dermal carotenoid level appeared inversely correlated with both these parameters. To the best of our knowledge, skin carotenoids have never been correlated to risk factors for cancer. The observed correlation between SCS and BMI and WC, two prognostic factors for breast cancer, appears to reflect the importance of all these parameters on this condition and allows SCS to be considered as a reliable additional tool to monitor at-risk individuals and/or effectiveness of interventions. In addition, our findings confirm the effectiveness of the RRS methodology to reveal body redox state even in patients whose antioxidant status is remarkably altered [26].

Breast cancer (BC) is one of the most common in women, with over 400,000 deaths worldwide every year [43]. Surgery, radiotherapy, chemotherapy, and/or hormone therapy are shown to be only partially effective in reducing morbidity, which emphasizes the importance of prevention. To this aim lifestyle factors, including diet, are considered fundamental [7–13]. Our work highlights the importance of a firm adhesion to a recommended dietary pattern and suggests a useful tool to help the patients' motivation. The SCS value has appeared an objective tool to evaluate the effectiveness of the dietary intervention in BC patients. Such a simple and noninvasive measurement may be useful to improve patient awareness of the importance of adhesion to healthy lifestyle and alimentary regimen to positively affect BC risk factors. In our experience these measurements allowed each patient to appraise the effectiveness of her own strains, helping her to understand the accuracy of the diet performed or whether corrections had to be made.
